# Typification of 23 names in *Eriobotrya* (Maleae, Rosaceae)

**DOI:** 10.3897/phytokeys.139.47967

**Published:** 2020-02-04

**Authors:** Bin-Bin Liu, Guang-Ning Liu, De-Yuan Hong, Jun Wen

**Affiliations:** 1 State Key Laboratory of Systematic and Evolutionary Botany, Institute of Botany, Chinese Academy of Sciences, Beijing 100093, China Institute of Botany, Chinese Academy of Sciences Beijing China; 2 Department of Botany, National Museum of Natural History, Smithsonian Institution, PO Box 37012, Washington, DC 20013-7012, USA National Museum of Natural History, Smithsonian Institution Washington United States of America; 3 College of Architecture and Urban Planning, Tongji University, Shanghai 200092, China Tongji University Shanghai China

**Keywords:** integrative systematics, lectotype, loquat, neotype, nomenclature, *Pyrus*, *Rhaphiolepis*, taxonomy

## Abstract

As part of a comprehensive systematic study on the genus *Eriobotrya* and its close relatives from the E & SE Asia, new typifications of 23 names are presented here, along with some nomenclatural notes of the names involved. We lectotypified 22 names including accepted names and synonyms. They are: *E.acuminatissima*, E.bengalensisvar.angustifolia; E.bengalensisf.intermedia, *E.brackloi*, E.brackloivar.atrichophylla, E.ellipticavar.petelotii, E.fragransvar.furfuracea, *E.glabrescens*, *E.grandiflora*, *E.henryi*, *E.oblongifolia*, *E.petiolata*, *E.platyphylla*, *E.poilanei*, *E.prinoides*, E.prinoidesvar.laotica, *E.salwinensis*, *E.serrata*, *E.stipularis*, *Hiptagecavaleriei*, *Photinialongifolia*, *Symplocosseguinii*. One neotype of *Photiniadubia* was also proposed in this study, and *E.pseudoraphiolepis* and *Mespiluscuila* were identified as superfluous names. In addition, we also summarized the typification of 18 names for taxonomic reference: *E.angustissima*, *E.balgooyi*, *E.condaoensis*, *E.×daduheensis*, *E.elliptica*, *E.fulvicoma*, *E.fragrans*, E.glabrescensvar.victoriensis, *E.hookeriana*, *E.latifolia*, *E.obovata*, *E.malipoensis*, *E.merguiensis*, *E.tengyuehensis*, *E.wardii*, *Mespilusbengalensis*, *Photiniadeflexa*, and *M.japonica*.

## Introduction

The application of names of taxa at the rank of family and below is determined by means of nomenclatural types (Shenzhen Code, Turland et al. 2018: Art. 7.1), and it is advisable to lectotypify those names with no type or sometimes even no original materials at all (Turland 2019). The type, usually a specimen or illustration, is the only entity permanently linked with the name. As one of the six principles in the International Code of Nomenclature for algae, fungi, and plants (Shenzhen Code, Turland et al. 2018), typification will pave the way for taxonomic stabilization of the names. The role of nomenclature in taxonomy was rightly expressed by Davis and Heywood (1963): “biologists must know what organisms they are working with before they can pass on information about them to other people – a function of taxonomy which makes stability of nomenclature an important consideration”.

Several groups of the tribe Maleae (Rosaceae) are well-known because of the numerous north temperate fruits and ornamentals, such as apples (*Malus* Mill. spp.), pears (*Pyrus* L. spp.), serviceberries (*Amelanchier* Medik. spp.), chokeberries (*Aronia* Medik. spp.), loquats (*Eriobotryajaponica* (Thunb.) Lindl.), and photinias (*Photinia* Lindl. spp.). Due to the polyphyly of *Sorbus* L. and frequent hybridization within and between genera in Maleae, Christenhusz et al. (2018) argued for merging all members of Maleae into *Pyrus* L., returning to the Linnaean concept, and 849 new names and new combinations in Maleae were proposed. This extreme taxonomic strategy will hardly be appropriate for this ecologically and economically important lineage. Although we listed all new combinations proposed by Christenhusz et al. (2018) in this study, we prefer to recognize the genera in a traditional sense for better communication between plant scientists and the genera publics (Crane et al. 2017; Liu et al. 2019). Due to the rapid radiation and frequent hybridization (Robertson et al. 1991; Campbell et al. 2007), limited markers from plastome and/or nuclear based on Sanger sequencing could not provide enough resolutions for resolving the phylogenetic relationships among the genera in Maleae. With the development of next-generation sequencing, large datasets could be obtained at an affordable cost. Resolving the phylogenetic and evolutionary questions in Maleae is getting much easier and the *Pyrus* s.l. concept might be premature. For example, Liu et al. (2019) used the whole chloroplast genome and entire nuclear ribosomal DNA obtained via genome skimming approach to clarify the generic delimitation between *Photinia* Lindl. and its morphological allies in Rosaceae (Liu et al. 2019); additionally, this method has also been successfully employed to resolve the systematic problems in other angiosperm lineages (Wen et al. 2018; Valcárcel and Wen 2019). Therefore, phylogenomics based on next/third-generation sequencing will provide an opportunity for clarifying the taxonomic and evolutionary problems in Maleae, especially *Eriobotrya* Lindl.

Loquats have been cultivated widely in the world as fruits and/or ornamentals. *Eriobotrya* consists of ca. 15–20 species, and is widely distributed from the Himalayas throughout continental southeast Asia to Japan and the islands of western Malesia (Kalkman 2004). The generic delimitation among the members of Maleae has been notoriously controversial for a long time, and efforts to resolve the intergeneric relationships have not been successful (Phipps et al. 1991; Campbell et al. 2007; Li et al. 2012; Lo and Donoghue 2012; Verbylaitė et al. 2006; Sun et al. 2018). As part of our integrative systematic studies of *Eriobotrya* and close relatives (Wen et al. 2017; Liu et al. 2020), it will be necessary to typify the taxa described under *Eriobotrya*.

## Materials and methods

We summarized the names listed in Tropicos, IPNI (International Plant Names Index), and the Plant List as the first step, and relevant literature has been consulted for the names treated in the present study. For the typification of each name summarized, all the relevant monographs and floras (e.g., Vidal 1965, 1968, 1970; Gu and Spongberg 2003) have been checked as well as the additional original literature publishing the names and combinations (listed below). The rules governing holotype recognization and lectotypification followed McNeill (2014), and Turland (2019), and Turland et al. (2018). Thanks to the world’s largest database of digitized plant specimens (JSTOR: Global Plant), we checked 158 images of specimens in the herbaria all around the world, including A, B, BK, BM, C, E, HBG, K, L, M, MO, MSC, NY, P, TCD, UPS, VNMN, and WU. The authors consulted the potential type material in the following herbaria: CDBI, HITBC, IBK, IBSC, KUN, PE, SN, SYS, SZ, US, and WUK (Index Herbariorum 2019).

## Typifications

***Eriobotrya*** Lindl., Trans. Linn. Soc. London 13: 96, 102 (1821). Lectotype, designated by L.K.G. Pfeiffer, Nom. 1: 1238 (1873): *Eriobotryajaponica* (Thunb.) Lindl. ≡ *Mespilusjaponica* Thunb.

**1. *Eriobotryaacuminatissima*** Nakai, J. Arnold Arbor. 5: 71. 1924. ≡ PhotinialuzonensisMerr.var.acuminatissima Merr. nom. nudum. Type: PHILIPPINES. Luzon: Panay Province, mt. Salibongbong Capiz, June 1919, *A. Martelino & G. Edano 35622* (**lectotype, designated here**: A[barcode 00026487]!; isolectotypes: BM[barcode BM000602127]!, L[barcode L0019714]!). [image of lectotype available at https://plants.jstor.org/stable/10.5555/al.ap.specimen.a00026487]

Nakai (1924) clearly cited one gathering (*A. Martelino & G. Edano 35622*) as the type in the protologue, however, he did not indicate the herbarium where the type has been deposited. We located three duplicates in A, BM, and L, all of them from the locality given in the protologue. According to Stafleu and Cowan (1981), most of Nakai’s specimens have been deposited at TI; a part of type material kept at A and P. However, we have not found any duplicate in TI. From 1923 to 1925, Nakai visited the principal botanical institutions in Europe and North America, including herbarium A (Hara, 1953), and *Eriobotryaacuminatissima* was published in 1924. We concluded that *E.acuminatissima* was described based on the specimens in herbarium A when he visited the herbarium A at Harvard University. We therefore designated the specimen at A as the lectotype, since it was annotated by Nakai: “Type; *Eriobotryaacuminatissima* Nakai”.

**2. *Eriobotryaangustissima*** Hook.f., Fl. Brit. India [J. D. Hooker] 2(5): 372. 1878. ≡ *Pyrusangustissima* (Hook.f.) M.F.Fay & Christenh., Global Fl. 4:95. 2018. Type: INDIA. Khasia. alt. 5000 ft., without date, *J.D. Hooker & T. Thomson s.n.* (lectotype, designated by Vidal 1965: 574): K[barcode K000758406]! “type”; isolectotype: BM[barcode BM000602192]!. Khasia. between [zugrung Thumblus], 26 July 1850, *s.coll. s.n.* (syntype K[barcode K000758404]!). Mooshye, Shrub 4 feet, 23 September 1850, *s.coll. s.n.* (syntype: K[barcode K000758405]!). [image of lectotype available at http://specimens.kew.org/herbarium/K000758406]

Vidal (1965) lectotypified this name without any explanation. We provide a nomenclature note for the lectotypification herein. In the protologue, Hooker (1878) provided the following locality when he described *Eriobotryaangustissima* in the Flora of British India: “Khashi Mts. alt. 5000 ft. *Simons*; Mooshye, and between Myrung and Nunklow, *Hook.f. & T.*”. These two gatherings are syntypes. Four potential syntypes have been located, and one is in BM and the other three in K. These three syntypes at K were mounted on one sheet. The first (K000758406) includes the only blooming specimens collected by J.D.H. & T.T., i.e. J.D. Hooker & T. Thomson, labeled “Hab. Khasia; [...]; alt. 5000 feet; Coll. J.D.H. & T.T.” without date. The collection in BM has the same label information. The second (K000758404) was collected from “Khasia. between [zugrung Thumblus]” without a collector, dated 26 July 1850. The third sheet (K000758405) collected from Mooshye was labeled as “Rosaceae?; Shrub 4 feet” also without a collector, dated 23 September 1850. The latter two specimens and the one in BM (BM000602192) are only vegetative branches and thus not optimal for lectotypification. Vidal (1965) designated the blooming specimen (K000758406) as the “type”, however, this specimen is actually a lectotype.

**3. *Eriobotryabalgooyi*** K.M.Wong & Ent, Pl. Ecol. Evol. 147(1): 136. 2014. Type: MALAYSIA. Sabah, Ranau District, Bukit Babi [Pig Hill] on the south-east side of Mount Kinabalu, 6°03'N, 116°36'E, 2000–2300 m, 25 May 1984, *J.H. Beaman et al. 9871* (holotype: K[barcode K000618095]!; isotype: MSC). Borneo. Sabah, Kinabalu Park, Mount Tambuyukon, main summit ridge, 2487 m elevation, 14 Apr. 2011, *Van der Ent et al. SNP 24531* (paratypes: SING, SNP), ibid., 2499 m elevation, 4 May 2011, *Van der Ent et al. SNP 25940* (paratypes: SING, SNP); near top of 2^nd^ summit, 2535 m elevation, 6 May 2011, *Van der Ent et al. SNP 26155* (paratypes: L, SING, SNP). [image of holotype available at http://specimens.kew.org/herbarium/K000618095]

**4. *Eriobotryabengalensis*** (Roxb.) Hook.f. var. ***angustifolia*** Cardot, Notul. Syst. (Paris) 3: 371. 1918. ≡ Eriobotryabengalensis(Roxb.)Hook.f.f.angustifolia (Cardot) J.E.Vidal, Adansonia, n.s. 5: 569. 1965. Type: CHINA. Yunnan: Hay-y près Lou-Lan, Pau Ngueou, 29 May 1907, *F. Ducloux 4719* (**lectotype, designated here**: P[barcode P02143256]!; isolectotype: P[barcode P02143257]!). [image of lectotype available at https://plants.jstor.org/stable/10.5555/al.ap.specimen.p02143256]

Cardot (1918) described Eriobotryabengalensisvar.angustifolia in his article “Rosacées Nouvelles D’extreme-Orient”. According to Stafleu and Mennega (1995), Cardot’s specimens are kept at P. We have located two sheets in P which represent duplicates from a homogeneous collection. The red tag on the sheet barcoded with P02143256 was obviously not made by Cardot, this sheet was not holotype (Art. 9.1, Turland et al. 2018). A further lectotypification thus is necessary. Furthermore, we have not found it to be published anywhere for the lectotypification (cf. Nakai 1924; Vidal 1965, 1968, 1970; Kuan and Yu 1974; Gu and Spongberg 2003). We followed the informal typification designated by the staff in herbarium P, and herein formally designate the sheet (P02143256) as the lectotype.

**5. *Eriobotryabengalensis*** (Roxb.) Hook.f. f. ***intermedia*** J.E.Vidal Adansonia, n.s. 5: 568. 1965. Type: MYANMAR. “In thicket on the western flank of the N’Maikha-Salween divide, east of Hpimaw. Lat. 26°N, alt. 10000 feet. East Upper Burmarh”, April 1919, *G. Forrest 17845* (**lectotype, designated here**: E[E00072976]!, isolectotype: E[E00072977]!, K). VIETNAM. “Prov. de Huê: Bach Ma, 1500 m”, *J.E. Vidal 35A* (paratype: P). VIETNAM. “Prov. de Huê: Bach Ma, 1500 m”, *J.E. Vidal 35B* (paratype: P). VIETNAM. “Prov. de Huê: Bach Ma, 1500 m”, *J.E. Vidal 35C* (paratype: P). [image of lectotype available at http://data.rbge.org.uk/herb/E00072976]

Vidal (1965) mentioned the locality in the protologue “CHINE. – Yun Nan: *Forrest 17845* (E, K). VIETNAM (Sud). – Région de Huê: *Vidal 35A, 35B, 35C* (P)” in his work “Notes sur quelques Rosacées asiatiques (III). Révision du genre *Eriobotrya* (Pomoideae)”. We found two specimens of *Forrest 17845* at E with an alternative locality “F. No. 17845. *Eriobotrya*. aff. *E.japonica*. Shrub of 20–25 feet. Flowers creamy-white. In thicket on the western flank of the N’Maikha-Salween divide, east of Hpimaw. Lat. 26°N, alt. 10000 feet. April 1919. East Upper Burmarh”. In addition, the printed “YUNNAN, WEST CHINA” was corrected as handwritten “East Upper Burmarh”, which may have occurred after Vidal published the name of Eriobotryabengalensisf.intermedia. These two duplicates (E00072976 and E00072977) have not been clearly cross-labeled as being part of the same specimen. It is necessary to select one of them as the lectotype. We designated the blooming specimen (E00072976) as the lectotype, as the other one only included a vegetative branch.

**6. *Eriobotryabrackloi*** Hand.-Mazz., Anz. Akad. Wiss. Wien, Math.-Naturwiss. Kl. 1922, lix. 102. ≡ Eriobotryacavaleriei(H.Lév.)Rehdervar.brackloi (Hand.-Mazz.) Rehder, J. Arnold Arbor. 13(3): 308. 1932. Type: CHINA. Kwangtung (Guangdong): In silva and austro-occid. jugi Tsatmukngao prope oppidum lienping ad bor.-or. urbis Kanton sita ad rivos, 800 m, substr. crystallino, 15, 27 July 1920, *R.E. Mell 659* (**lectotype, designated here**: WU[barcode 0059394]!; isolectotype A[barcode 00026469]!). [image of lectotype available at https://plants.jstor.org/stable/10.5555/al.ap.specimen.wu0059394]

Handel-Mazzetti (1922) provided the following locality in the protologue when he described *Eriobotryabrackloi*: “Prov. Kwangtung: In silva ad austro-occid. jugi Tsatmukngao prope oppidum Lienping ad bor.-or. urbis Kanton sita ad rivos, 800 m, substr. crystallino, leg. 15. 27. VII. 1920 Mell (Pl. M. S. Nr. 659)”. According to Stafleu and Cowan (1979), the main set of specimens and types are kept at W and WU, although there is original material in other herbaria too. We located original material at WU and A, both of them matching the locality mentioned above. We designate the specimen at WU (barcode 0059394) where Handel-Mazzetti worked as the lectotype of the name *E.brackloi*, with the one in A (barcode 00026469) as isolectotype.

**7. *Eriobotryabrackloi*** Hand.-Mazz. var. ***atrichophylla*** Hand.-Mazz., Anz. Akad. Wiss. Wien, Math.-Naturwiss. Kl. 1922, lix. 103. Type: CHINA. Hunan: austro-occ.: In monte Yün-scha prope urbem Wukang, in silva elata frondosa umbrosa. alt. 950 m, 6 June 1918, *H.F. von Handel-Mazzetti 12032* (**lectotype, designated here**: WU[barcode 0059395]!, isolectotype: A[barcode 00026471]!). ibidem, alt. 1300 m, 9 June 1918, *H.F. von Handel-Mazzetti 12060* (syntypes: A[barcode 00026470]!, WU[barcode 0059396]!, WU[barcode 0059397]!). [image of lectotype available at https://plants.jstor.org/stable/10.5555/al.ap.specimen.wu0059395]

Handel-Mazzetti (1922) described Eriobotryabrackloivar.atrichophylla in his article “Sitzung der mathematisch-naturwissenschaftlichen Klasse” and cited two gatherings (syntypes) in the protologue, “950 (Nr. 12.032) et 1300 (Nr. 12.060)”, from which the lectotype can be selected. Both of the gatherings match perfectly the locality in the protologue; we choose one duplicate from *H.F. von Handel-Mazzetti 12032*. We located two sheets of *H.F. von Handel-Mazzetti 12032* in WU and A. According to Stafleu and Cowan (1979), the main set of original material are kept in WU and W, so we designated WU(barcode 0059395) as the lectotype, with A(barcode 00026471) as the isolectotype.

**8. *Eriobotryacondaoensis*** X.F.Gao, Idrees & T.V.Do, Phytotaxa 365(3): 290. 2018. Type: VIETNAM. Ba Ria-Vung Tau Province: Con Dao National Park, growing on the slope of hill under tropical evergreen forest, 20m, 8°41'30"N, 106°38'00"E, 21 March 2017, *T.V.Do VNMN_CN 633* (holotype: VNMN!; isotype CDBI!).

**9. *Eriobotrya×daduheensis*** H.Z.Zhang ex W.B.Liao, Q.Fan & M.Y.Ding, Phytotaxa 212(1): 97. 2015. Type: CHINA. Sichuan: Hanyuan County, Dashu Town, Xinmin Village, Mt. Shizishan, in the forest edge at the foot of the mountain, 970 m, 29°17'48.18"N, 102°39'44.94"E, 19 December 2007, *Q. Fan 9292* (holotype: SYS[barcode 190936]!; isotypes [SYS!, IBSC!]).

**10. *Eriobotryaelliptica*** Lindl., Trans. Linn. Soc. London 13(1): 102 (1821). ≡ *Eriobotryaelliptica* (Lindl.) Hook.f. & Thomson, Fl. Brit. India [J. D. Hooker] 2(5): 372. 1878. ≡ *Cotoneasterellipticus* (Lindl.) Hort ex Loudon, Encyc. Pl. 1208.≡ *Pyruselliptica* (Lindl.) M.F.Fay & Christenh. Global Fl. 4:102. 2018. Type: NEPAL. Narainhetty. 1 February 1803, *F. Buchanan-Hamilton s.n.* (holotype: BM[barcode BM000521994]!). [note 1] [image of holotype available at https://data.nhm.ac.uk/object/d27f7005-730e-4ed6-bc6c-e140e89bfd5d/1566345600000]

= *Mespiluscuila* Buch.-Ham. ex D.Don, Prodr. Fl. Nepal. 238. 1825. nom. superfl. [note 2]

Note 1: Lindley (1821) described *Eriobotryaelliptica* in his work “Observation on the natural group of plants called Pomaceae” and provided the following locality in the protologue: “Hab. ad Narainhetty, *Buchanan* (*v. s. sp. Herb. Lambert*)”. The specimen(s) used by the original author was indicated as part of Herb. Lambert, however, some specimens of that collection had been purchased by National History Museum (BM) (Miller 1970). We located one specimen in BM labeled as “Nepaul. Dr. Buchanan; Narainhetty. 1^st^ Feb. 1803” which was perfectly in accordance with the protologue. So this specimen could be the original material used by Lindley when he published the name of *E.elliptica* and must be the holotype which was indicated by Vidal (1965).

Note 2: Don (1825) described *Mespiluscuila* with the following locality: “Hab. ad Nrainhetty Nepalensium. *Hamilton*”. This specimen was designated as the type of *Eriobotryaelliptica* Lindl. (1821). *M.cuila* thus was a nomenclaturally superfluous name when it was published (Art. 52.1, Turland et al. 2018).

**11. *Eriobotryaelliptica*** Lindl. var. ***petelotii*** J.E.Vidal, Adansonia sér. 2, 5: 552. 1965. Type: VIETNAM. “prov. de Lao Kay, Chapa, 1500 m”, January 1929, *M. Pételot s.n.* (**lectotype, designated here**: P[barcode P02143261]!; isolectotype: P[barcode P02143262]!). [image of lectotype available at https://plants.jstor.org/stable/10.5555/al.ap.specimen.p02143261]

In the protologue of Eriobotryaellipticavar.petelotii, Vidal (1965) mentioned: “Vietnam (Nord), prov. Lao Kay, Chapa, 1500 m, en fleurs, janv. 1929, *Pételot s.n.* (P)”. We located two syntypes kept at P, from which the lectotype could be chosen. These two specimens both have complete information in accordance with the protologue. We designated the sheet P[barcode P02143261] as the lectotype, as it has been labeled with a red printed tag “TYPE”.

**12. *Eriobotryafulvicoma*** Chun ex W.B.Liao, F.F.Li & D.F.Cui, Ann. Bot. Fenn. 49(4): 264. 2012. Type: CHINA. Guangdong: Xinyi County, Dawuling Natural Reserve, 45 m, 28 April 1932, *Z. Huang 32257* (holotype: WUK[barcode 0109531]!; isotypes: IBK[barcode 00060958]!, IBK[barcode 00060976]!, IBSC[barcode 0298975]!, KUN[barcode 0116268]!, PE[barcode 00799336]!, SZ[barcode 00194329]!). *s. loc.*, 23 April 1932, *Z. Huang 32174* (paratypes: IBSC[barcode 0298973]!, IBK[barcode 00060963]!, IBK[barcode 00060975]!, KUN[barcode 0116267]!, PE[barcode 00799340]!, WUK[barcode 0110493]!, SN[barcode 007751]!, SZ[barcode 00194441]!). *s. loc.*, *Z. Huang 29869* (paratype: IBSC).

**13. *Eriobotryafragrans*** Champ., Hooker’s J. Bot. Kew Gard. Misc. 4: 80. 1852. ≡ *Pyruswilliamtelliana* M.F.Fay & Christenh. Global Fl. 4:126. 2018. Type: CHINA. Hong Kong: Mt. Victoria, *J.G. Champion s.n.* (lectotype, designated by Vidal: 557. 1965: K[barcode K000758384]! “type”). [image of lectotype available at http://specimens.kew.org/herbarium/K000758384]

The name *Eriobotryafragrans* was published in Bentham’s paper in 1852, however, it was ascribed to “Champ.”, although the description was described by Bentham. The name is therefore cited as *E.fragrans* Champ. instead of *E.fragrans* Champ. ex Benth. as indicated in Tropicos and IPNI.

**14. *Eriobotryafragrans*** Champ. var. ***furfuracea*** J.E.Vidal, Adansonia sér. 2, 5: 557. 1965. Type: VIETNAM (Sud-Annam). Nha Trang: Massif du Hon Ba, 1000–1500 m, en fleurs, 5 September 1918, *A. Chevalier 38893* (**lectotype, designated here**: P[barcode P02143263]!; isolectotypes: A[barcode 00026481]!, C[barcode C10017884]!, K[barcode K000758407]!, L[barcode L0019413]!, P[barcode P02143264]!, P[barcode P02143265]!, P[barcode P02143266]!). [image of lectotype available at https://plants.jstor.org/stable/10.5555/al.ap.specimen.p02143263]

According to Stafleu and Cowan (1976), Chevalier’s specimens were deposited in P and PC, although there is original material in other herbaria too. We located eight original specimens in A, C, K, L, and P, and all of them are in accordance with the locality in the protologue “Vietnam (Sud), prov. Nha Trang, massif du Hon Ba, 1000–1500 m”. The author (Vidal) designated the specimens in P as type, however, three sheets were located in herbarium P. We designate the specimen (P[barcode P02143263]) with red printed tag “TYPE” as the lectotype.

**15. *Eriobotryaglabrescens*** J.E.Vidal, Adansonia sér. 2, 5: 554. 1965. ≡ *Pyrusserpentae* M.F.Fay & Christenh. Global Fl. 4:121. 2018. Type: MYANMAR. Kachin State: “N. Birmanie, Triangle, Hkinlum village, 2500 m, en fleurs”, 4 April 1953, *F. Kingdon-Ward 20616* (**lectotype, designated here**: BM[barcode BM000602189]; isolectotypes: A[barcode 00026482]!, E[00011336]!). [image of lectotype available at https://plants.jstor.org/stable/10.5555/al.ap.specimen.bm000602189]

Vidal (1965) designated the gathering *F. Kingdon-Ward 20616* (BM, E) as type. We located three original specimens in A, BM, and E, all of them matched perfectly with the locality in the protologue. We designate the specimens kept at BM with a red printed tag “TYPE” as the lectotype.

**16. *Eriobotryaglabrescens*** J.E.Vidal var. ***victoriensis*** J.E.Vidal, Adansonia sér. 2, 5: 555. 1965. Type: MYANMAR. KachinState: “Birmanie centrale, Mt Victoria, 3000 m, en fleurs”, 2 April 1956, *F. Kingdon-Ward 21915* (holotype: BM[BM000602190]!). Mt Victoria, *F. Kingdon-Ward 22828* (paratype: BM). [image of holotype available at https://plants.jstor.org/stable/10.5555/al.ap.specimen.bm000602190]

Vidal (1965) designated *Kingdon-Ward 21915* (BM) as type, and only one specimen with a handwritten tag was found in BM. So, the specimen barcoded with BM000602190 is the holotype.

**17. *Eriobotryagrandiflora*** Rehder & E.H.Wilson, Pl. Wilson. (Sargent) 1(2): 193. 1912. ≡ Eriobotryadeflexa(Hemsl.)Nakaivar.grandiflora (Rehder & E.H.Wilson) Nakai J. Arnold Arbor. 5(2): 72. 1924. Type: CHINA. Western Szech’uan (Sichuan): alt. 1600m, May 1904, *Veitch Exped. 3506* (**lectotype, designated here**: A[barcode 00026472]!; isolectotypes: A[barcode 00026473]!, BM[barcode BM000602187]!, HBG[barcode HBG511040]!, K[barcode K000758386]!, P[barcode P02143267]!). Mupin, alt. 1300 m, October 1908, *s.coll. 2999* (paratype). [image of lectotype available at https://plants.jstor.org/stable/10.5555/al.ap.specimen.a00026472]

Rehder and Wilson (1912) mentioned the following locality when describing *Eriobotryagrandiflora*: “Western Szech’uan: without precise locality, alt. 1600 m, May 1904 (Veitch Exped. No. 3506, type)”. The specimens of “Plantae Wilsonianae” were mainly deposited in A (Rehder and Wilson 1912). We have not found potential original material numbered as *Veitch Exped. 3506* among the available collections at A. Nonetheless, we located two specimens of *E.H. Wilson 3506* with the following description: “Coll. E.H. Wilson, (For James Veitch & Sons). Western China. Type. No. 3506. ...”. The locality, date, and morphology are perfectly in accordance with the description in the protologue. We located six sheets of *E.H. Wilson 3506* in A, BM, HBG, K, and P, from which the lectotype could be chosen. We chose the sheet kept at herbarium A as the lectotype, where Rehder & Wilson worked. Although both duplicates are from a single gathering (A00026472 and A00026473) and annotated with “Type”, they have not been cross-labeled as being part of the same specimen and do not bear a single, original label in common (Art. 9.3, Turland et al. 2018). It is necessary to select one of them as the lectotype. The sheet (A [barcode 00026472]) was designated as the lectotype, since it is in a better condition.

**18. *Eriobotryahenryi*** Nakai, J. Arnold Arbor. 5: 70. 1924. ≡ *Pyrushenryi* (Nakai) M.F.Fay & Christenh. Global Fl. 4:106. 2018. Type: CHINA. Yunnan: Szemao (Simao), 1900, *A. Henry 13018* (**lectotype** (selected by Vidal 1965: 562, first step “type”; **second step, designated here**): A[barcode 00026474]!; isolectotypes: K[barcode K000758388]!, NY[barcode 00436209]!). ibidem, *A. Henry 11644A* (syntypes: A[barcode 00026475]!, MO[barcode MO-176737]!). ibidem, A. *Henry 11644* (syntype: A[barcode 00063057]!). [image of lectotype available at https://plants.jstor.org/stable/10.5555/al.ap.specimen.a00026474]

Nakai (1924) described *Eriobotryahenryi* on the basis of three gatherings “*A. Henry*, nos. 13018, 11644, 11644A” which were syntypes. Vidal (1965) wrote “*Henry 13018* (type)”, thereby designating the gathering *Henry 13018* as the lectotype [first-step]. We located two specimens of this gathering in A and K, from which the lectotype could be chosen. The specimen deposited in A is designated as the lectotype [second-step] as it has an autograph of Nakai “*Eriobotryahenryi* Nakai” and Vidal’s handwriting selecting the lectotype [first-step] “lectotype; *Eriobotryahenryi* Nakai; J. Vidal 6/1964”.

**19. *Eriobotryahookeriana*** Decne., in Nouv. Arch. Mus. Par. Ser. I, x. 146. 1874. ≡ *Pyrushookeriana* (Decne.) M.F.Fay & Christenh. Global Fl. 4: 107. 2018. Type: INDIA. Sikkim: 10 August 1862, *T. Anderson 490* (lectotype, designated by Vidal: 563: 1965: P[barcode P02143268]! “type”; isolectotype: A[barcode 00026483]!). Sikkim, *J.D. Hooker & T. Thomson 579* (syntypes: K[barcode K000758396]!, K[barcode K000758397]!, P[barcode P02143269]!). [image of lectotype available at https://plants.jstor.org/stable/10.5555/al.ap.specimen.p02143268]

**20. *Eriobotryalatifolia*** Hook.f., Fl. Brit. India [J. D. Hooker] 2(5): 370. 1878. ≡ *Pyrusherae* M.F.Fay & Christenh. Global Fl. 4:106. 2018. Type: MYANMAR. Moalmayne; on Thoung Gyne, alt. 5000 ft., *T. Lobb s.n.* (holotype: K[barcode K000758400]!). [image of holotype available at http://specimens.kew.org/herbarium/K000758400]

**21. *Eriobotryamalipoensis*** K.C.Kuan, Acta Phytotax. Sin. viii. 231. 1963. ≡ *Pyrusmalipoensis* (K.C.Kuan) M.F.Fay & Christenh., Global Fl. 4:111. 2018. Type: CHINA. Yunnan: Malipo County, Hwang-jin-yinn, 1200 m, 21 January 1940, *C.W. Wang et al. 86318* (holotype: PE[barcode 00004573]!; isotypes: IBSC[barcode 0299391]!, KUN[barcode 0116367]!). ibidem, 13 January 1940, *C.W. Wang 83170* (paratypes: HITBC[barcode 016193]!, IBK[barcode 00366990]!, IBSC[barcode 0299392]!, KUN[barcode 0116366]!, PE[barcode 00799655]!). ibidem, 9 November 1947, *K.M. Feng 13123* (paratypes: KUN[barcode 0116370]!, PE[barcode 00004572]!).

**22. *Eriobotryamerguiensis*** J.E.Vidal, Adansonia sér. 2, 5: 563. 1965. ≡ *Pyrusmerguiensis* (J.E.Vidal) M.F.Fay & Christenh. Global Fl. 4:112. 2018. Type: MYANMAR. “Birmanie, Mergui, Mout Myinmolekat, 1200 m, en fruits”, 17 January 1930, *R.N. Parker 3098* (holotype: K[K000758399]!). [image of holotype available at http://specimens.kew.org/herbarium/K000758399]

**23. *Eriobotryaoblongifolia*** Merr. & Rolfe, Philipp. J. Sci., C 3: 102. 1908. Type: PHILIPPINES. Mindanao. Misamis: Mount Malindang, May 1906, *E.A. Mearns & W.J. Hutchinson 4680* (**lectotype, designated here**: NY[barcode 00436215]!; isolectotype: US[barcode 00097490]!). [image of lectotype available at https://plants.jstor.org/stable/10.5555/al.ap.specimen.ny00436215]

Merrill (1908) mentioned one gathering (*E.A. Mearns & W.J. Hutchinson 4680*) as type. We located two original specimens kept in NY and US, from which the lectotype could be chosen. According to Stafleu and Cowan (1981), Merrill’s type and material are deposited in A, FH, NY, PNH, and UC. We, therefore, designate the sheet deposited in NY as the lectotype.

**24. *Eriobotryaobovata*** W.W.Sm., Notes Roy. Bot. Gard. Edinburgh 10: 29. 1917. ≡ *Pyrusobovata* (W.W.Sm.) M.F.Fay & Christenh. Global Fl. 4:114. 2018. Type: CHINA. Yunnan: in the vicinity of Yunnanfu, *E.E. Maire 2450* (holotype: E[barcode E00011331]!; isotypes: E[barcode E00284668]!, K[barcode K000758390]!). [image of holotype available at http://data.rbge.org.uk/herb/E00011331]

Smith (1917) provided the following locality in the protologue when describing *Eriobotryaobovata*: “China: – In the vicinity of Yunnanfu, Yunnan. Maire. No. 2450. In Herb. Edin.” We located three original specimens in E and K, two of them kept in E. It should be noted that Smith (1917) indicated the specimens deposited in E as type, and the duplicate (E00011331) annotated “*Eriobotryaobovata* W.W.Sm. Type” with no indication of type on the other specimen. These two specimens have not been cross-labeled as being part of a same specimen. Neither have they born a single, original label in common (Art. 8.3, Turland et al. 2018). The duplicate (E00011331), therefore is the holotype and the other one in E (E00284668) the isotype.

**25. *Eriobotryapetiolata*** Hook.f., Fl. Brit. India [J. D. Hooker] 2(5): 370. 1878. ≡ *Pyruspetiolata* (Hook.f.) M.F.Fay & Christenh. Global Fl. 4:115. 2018. Type: Sikkim, 9000 ft, *J.D. Hooker s.n.* (**lectotype, designated here**: K[barcode K000758394]). East Himalaya, *W. Griffith 2085* (syntype: P[barcode P02143222]!). East Himalaya, *W. Griffith 2086* (syntypes: P[barcode P02143223]!, P[barcode P02143224]!). [image of lectotype available at http://specimens.kew.org/herbarium/K000758394]

Hooker (1878) described *Eriobotryapetiolata* with the following locality in the protologue in the Flora of British India: “Eastern Himalaya; Sikkim, alt. 5–9000 ft. Bhotan at Tongsa, *Griffith*.”

According to Stafleu and Cowan (1979), the main set of Hooker’s type and material has been deposited in K, although some of them are in MANCH and E too. We located one specimen in K with two labels, “Sikkim mts; Jayl? 9000 ft” and “[...] Hat. Sikkim; [...]; alt. 9000 feet; coll. J. D. H.”. This locality fitted with the locality provided by Hooker: “Sikkim, alt. 5–9000 ft.”, therefore, we designate this sheet K[barcode K000758394] as the lectotype of the name *E.petiolata*. We also located two gatherings from herbarium P collected by Griffith in the East Himalaya, *Griffith 2085* (P02143222) and *Griffith 2086* (P02143223, P02143224). These three specimens partially matched with the locality in the protologue: “Eastern Himalaya; [...] *Griffith*”. So, these three specimens deposited in P are syntypes.

**26. *Eriobotryaplatyphylla*** Merr., Brittonia iv. 80. 1941. ≡ *Pyrusplatyphylla* (Merr.) M.F.Fay & Christenh., Global Fl. 4: 116. 2018. Type: MYANMAR. Upper Burma: hills east of Fort Hertz, 8 December 1931, *F. Kingdon-Ward 10205* (**lectotype, designated here**: A[barcode 00026485]!; isolectotypes: A[barcode 00026484]!, BM[barcode BM000602191]!). [image of lectotype available at https://plants.jstor.org/stable/10.5555/al.ap.specimen.a00026485]

Merrill (1941) cited one gathering, *F. Kingdon-Ward 10205*, as the type when describing *Eriobotryaplatyphylla*. We located three original specimens, two in A and one in BM. Merrill’s type and material have been deposited in A, FH, NY, PNH, and UC. It is necessary to select one of two sheets in A as the lectotype, therefore, we designate the sheet (A[barcode 00026485]) with a blooming branch as the lectotype.

**27. *Eriobotryapoilanei*** J.E.Vidal, Adansonia sér. 2, 5: 557 (1965). ≡ *Pyruspoilanei* (J.E.Vidal) M.F.Fay & Christenh., Global Fl. 4: 116. 2018. Type: VIETNAM. Haut Donnai: Annam, Canton de Laouan Délégation de Djiriing, alt. 1200 m, 5 June 1933, *E. Poilane 22591* (**lectotype, designated here**: P[barcode P02143226]!; isolectotypes: C[barcode C10017885]!, L[barcode L0019414]!, P[barcode P02143227]!, P[barcode P02143228]!). [image of lectotype available at https://plants.jstor.org/stable/10.5555/al.ap.specimen.p02143226]

Vidal (1965) cited the gathering (*E. Poilane 22591*) deposited in P as the type. We located five sheets of this gathering in C, L, and P, three of them kept in the herbarium P. We designate the sheet (P02143226) as the lectotype, since it has a printed red tag “TYPE”.

**28. *Eriobotryaprinoides*** Rehder & E.H.Wilson, Pl. Wilson. (Sargent) 1(2): 194. 1912. ≡ *Pyrusprinoides* (Rehder & E.H.Wilson) M.F.Fay & Christenh., Global Fl. 4: 116. 2018. Type: CHINA. Yunnan: Mengtze (Mengzi), alt. 1500 m, *A. Henry 9878* (**lectotype, designated here**): A[barcode 00026476]!; isolectotypes: A[barcode 00026478]!, B[barcode B 10 0295749]!, E[barcode E00011334]!, K[barcode K00075839, excluding the fruiting branch]!, MO[barcode MO-176739]!, NY[barcode 00436210, excluding the fruiting branch]!, NY[barcode 00436211]!, NY[barcode 00436212]!, US[barcode 00097491, excluding the fruiting branch]!). *s. loc.*, *A. Henry 9878* (paratypes: A[barcode 00026477]!, E[barcode E00284667]!, K[barcode K00075839, excluding the flowering branch]!, MO[barcode MO-176738]!, NY[barcode 00436210, excluding the flowering branch]!, US[barcode 00097491, excluding the flowering branch]!). West Szech’uan: Tung Valley, on cliffs, alt. 800 m, May 1904, *Veitch Exped. 3507* (paratypes: A[00137409]!, A[00137410]!) [image of lectotype available at https://plants.jstor.org/stable/10.5555/al.ap.specimen.a00026476]

Rehder and Wilson (1912) cited two gatherings (*A. Henry 9878* & *Veitch Exped. 3507*), in which the former was mentioned as type. We found 14 sheets of *A. Henry 9878* among the available collections at A, B, E, K, MO, NY, and US, however, some of them are admixtures of flowering and fruiting branches collected by the same collector at different times. The new species described in “Plantae Wilsonianae” were considered to be on the basis of the specimens deposited in A. Three sheets of the type gathering were located in A, in which two of them are flowering branches and the remaining one is a fruiting branch. We designate one of the flowering specimens (A[barcode 00026476]) as the lectotype, since it is in better condition.

**29. *Eriobotryaprinoides*** Rehder & E.H.Wilson var. ***laotica*** J.E.Vidal, Adansonia sér. 2, 5: 573 (1965). Type: LAOS. Xièng Khouang: 1200 m, en fleurs, 3 November 1920, *M. Poilane 2243* (**lectotype, designated here**: P[barcode P02143229]!; isolectotypes: P[barcode P02143230]!, P[barcode P02143231]!). [image of lectotype available at https://plants.jstor.org/stable/10.5555/al.ap.specimen.p02143229]

Vidal (1965) cited one gathering (*M. Poilane 2243*) as the type in the protologue, however, we located three original specimens among the available collections in P, from which the lectotype could be chosen. The sheet (P02143229) with a red printed tag “TYPE” is designated as the lectotype herein.

**30. *Eriobotryasalwinensis*** Hand.-Mazz., Symb. Sin. Pt. VII. 475. 1933. ≡ *Pyrussalwinensis* (Hand.-Mazz.) M.F.Fay & Christenh., Global Fl. 4: 120. 2018. Type: CHINA. Yunnan: Im str. Laubwalde des birm. Mons. am Ufer des Salwin um Tschamutong von Sijitong bis unter Tjiontson, Phyllit und kristallinischer Kalk, 1625–1700 m, 13 July & 17 August 1916, *H.F. von Handel-Mazzetti 9573* (**lectotype, designated here**: WU[barcode 0059392]!; isolectotype: A[barcode 00026480]!). Mekong – Salwin-Kette, in Gebüschen an Bächen der Seitentäler am 28°12', 2420 m, *G. Forrest 16400* (paratype: WU). [image of lectotype available at https://plants.jstor.org/stable/10.5555/al.ap.specimen.wu0059392]

Handel-Mazzetti (1933) cited two gatherings in which *H.F. von Handel-Mazzetti 9573* was designated as the type in the protologue when describing *Eriobotryasalwinensis*. According to Stafleu and Cowan (1979), Handel-Mazzetti’s type material was mainly deposited in W & WU. We designate the specimen (WU[barcode 0059392]) as the lectotype herein with the duplicate kept in A as the isolectotype.

**31. *Eriobotryaserrata*** J.E.Vidal, Adansonia sér. 2, 5: 558. 1965. ≡ *Pyrusserrata* (J.E.Vidal) M.F.Fay & Christenh., Global Fl. 4: 121. 2018. Type: LAOS. Xièng Khouang: Ban Na Poun, 1200 m, en fleurs, 19 November 1920, *E. Poilane 2345* (**lectotype, designated here**: P[barcode P02143235]!; A[barcode 00026486]!, L[barcode L0019415]!, P[barcode P02143237]!, P[barcode P02143236]!). [image of lectotype available at https://plants.jstor.org/stable/10.5555/al.ap.specimen.p02143235]

Vidal (1965) mentioned the following typification in the protologue: “*Poilane 2345* (P)”. We located three syntypes in P, from which the lectotype could be chosen, and two other duplicates were also found in A and L. Vidal’s type material is commonly considered to be kept in P. We designate the sheet (P02143235) with a red printed tag “TYPE” as the lectotype.

**32. *Eriobotryastipularis*** Craib, Bull. Misc. Inform. Kew 1929(4): 109. 1929. ≡ *Pyrusstipularis* (Craib) M.F.Fay & Christenh., Global Fl. 4: 122. 2018. Type: THAILAND. Siam: Satul, Adang, 1500 m, on rocky ridge, 16 January 1928, *A.F.G. Kerr 14125* (**lectotype, designated here**: K[barcode K000758408]!; isolectotypes: ABD, BK[barcode 257292]!, BM, K[barcode K000758408]!, TCD[barcode TCD0016606]!). [image of lectotype available at http://specimens.kew.org/herbarium/K000758408]

Craib (1929) mentioned the following locality in the protologue: “Satul, Adang, 500 m, on the rocky ridge, *Kerr* 14125”. Vidal (1970) listed three herbaria, ABD, BM, and K, in which the type has been deposited, however, we located only four original specimens in BK, K, and TCD and did not find any duplicates in ABD and BM. According to Stafleu and Cowan (1976), Craib’s type material has been deposited in K and WRSL. We designate the sheet (K000758408) as the lectotype, since it has a complete inflorescence.

**33. *Eriobotryatengyuehensis*** W.W.Sm., Notes Roy. Bot. Gard. Edinburgh 10: 30. 1917. ≡ *Pyrustengyuehensis* (W.W.Sm.) M.F.Fay & Christenh., Global Fl. 4:123. 2018. Type: CHINA. Yunnan: Shweli-Salween divide, Lat. 25°5'N, alt. 7000 ft., tree of 40–60 ft., flowers creamy-yellow, open forests, May 1913, *G. Forest 9857* (lectotype, designated by Vidal: 571. 1965: E[barcode E00011333]!). Yunnan: Machang-Kai Valley, north of Tengyueh, Lat. 25°20'N, alt. 6000–7000 ft., shrub of 25–40 ft., flowers creamy-yellow, fragrant, in thickets, April 1913, *G. Forest 9847* (syntype: BM[barcode BM000602188]!). [image of lectotype available at http://data.rbge.org.uk/herb/E00011333]

**34. *Eriobotryawardii*** C.E.C.Fisch., Bull. Misc. Inform. Kew 1929(6): 205. 1929. ≡ *Pyrusalabaster* M.F.Fay & Christenh., Global Fl. 4:94. 2018. Type: MYANMAR. Namkiu Mountains. Valley of the Sheinghku, 6000–7000 ft., in flower in October, *F. Kingdon-Ward 7618* (holotype: K[barcode K000758392]!; isotype: A[barcode 00026488]! image of the holotype with a small fragment of inflorescence). [image of holotype available at http://specimens.kew.org/herbarium/K000758392]

**35. *Hiptagecavaleriei*** H.Lév., Repert. Spec. Nov. Regni Veg. 10: 372. 1912. ≡ *Eriobotryacavaleriei* (H.Lév.) Rehder, J. Arnold Arbor. 13: 307. 1932. ≡ *Pyrusathenae* M.F.Fay & Christenh., Global Fl. 4:96. 2018. Type: CHINA. Kouy-Tcheou (Guizhou): Pin-fa, montagne en pente, 20 May 1907, *J. Cavalerie 3220* (**lectotype, designated here**: E[barcode E00011330]!; isolectotypes: A[barcode 00055347]!, E[barcode 00284669]!, K[barcode K000758387]!, P[barcode P02143258]!, P[barcode P02143259]!). [image of lectotype available at https://plants.jstor.org/stable/10.5555/al.ap.specimen.p02143258]

Léveillé (1912) did not provide any typification information in the protologue, but mentioned it in his “Flore du Kouy-Tchéou” with the following information: “Pin-fa, montagne en pente, mai 1907, (Cavalerie 3220)”. According to Stafleu and Cowan (1979), all of Léveillé’s type specimens have been purchased by E in 1919, including the collections by J. Cavalerie. We located six duplicates among the available collections in A, E, K, and P, two of which have been deposited in E. Although all of Léveillé’s names have been comprehensively reviewed by Lauener (1983), he did not provide the typification information. Since the sheet (E00011330) has been annotated by Léveillé “*Hiptagecavaleriei* Levl..” and is in a good condition, we designate this sheet as the lectotype, the other five ones as the isolectotypes.

**36. *Mespilusbengalensis*** Roxb., Hort. Bengal. 38; Fl. Ind. ii. 510. 1832. ≡ *Eriobotryabengalensis* (Roxb.) Hook.f., Fl. Brit. India [J. D. Hooker] 2(5): 371. 1878. Type: INDIA. 1824, *N. Wallich 668.2* (neotype, designated by Vidal: 567: 1965: K[K001111550]! “lectotype”; isoneotype: P[barcode P02143255]!). [image of neotype available at http://specimens.kew.org/herbarium/K001111550]

Roxburgh (1832) did not cite any typification except of a locality in the protologue: “[...], a native of Chittagong, [...]”. According to Stafleu and Cowan (1983), Roxburgh’s main collection should be at K. We have not found any potential original material among the available collections at K, and this was also indicated by Vidal (1965, 1968). Although some of Roxburgh’s names have been validated and typified (Robinson 1912; Forman 1997; Turner 2013), *Mespilusbengalensis* has never been typified. Vidal, therefore, selected one specimen, *Wallich 668.2*, from K as the neotype. It should be noted that Vidal (1965) designated it as the lectotype, but later corrected this to neotype in his Flore du Cambodge, du Laos et du Vietnam (Vidal 1968).

**37. *Photiniadubia*** Lindl., Trans. Linn. Soc. London 13(1): 104, t. 10. 1821. ≡ *Eriobotryadubia* Decne., in Nouv. Arch. Mus. Par. Ser. I, x. (1874) 145. Type: NEPAL. *N. Wallich 668.1* (**neotype, designated here**: K[barcode K001111549]!; isoneotypes: BM[barcode 000521995(BM)]!, E[barcode E00011335]!). [image of lectotype available at http://specimens.kew.org/herbarium/K001111549]

Lindley (1821) provided the following locality in the protologue when describing *Photiniadubia*: “Hab. in Nepalia, *Wallich* (*v. s. sp. Herb. Banks et Lambert.*)”. But we have not found any potential original material which was in accordance with the locality in the protologue among the available collections. According to Stafleu and Cowan (1976, 1979), Bank’s type material is kept at CGE and K, and Lambert’s herbarium was sold to many herbaria including K and BM after his death (Miller 1970). We selected one specimen (K001111549) at K as the neotype with the following tag “668 *Photiniadubia* Lindl. Herb. 1824; 1 Nepalia 1821; [...]”, which partially fitted with the locality in the protologue. The duplicate in BM is an isoneotype.

**38. *Photiniadeflexa*** Hemsl., in Ann. Bot. ix. 153. 1895. ≡ *Eriobotryadeflexa* (Hemsl.) Nakai, Bot. Mag. (Tokyo) 30: 18, in adnot. 1916. Type: CHINA. Formosa (Taiwan): Bankinsing, May 1894, *A. Henry 498* (lectotype, designated by Vidal 1965: 566: K[barcode K000758389]! “type”; isolectotype: A[barcode 00026740]!). [image of lectotype available at http://specimens.kew.org/herbarium/K000758389]

**39. *Photinialongifolia*** Decne., in Nouv. Arch. Mus. Par. Ser. I, x. 142. 1874. ≡ *Eriobotryalongifolia* (Decne.) Hook.f., Fl. Brit. India [J. D. Hooker] 2(5): 370. 1878. Type: BANGLADESH. East Bengal. Mishmi Hills, *W. Griffith 2093* (**lectotype, designated here**: P[barcode P02143220]!; isolectotype: K[barcode K000758398]!). [image of lectotype available at https://plants.jstor.org/stable/10.5555/al.ap.specimen.p02143220]

Decaisne (1874) provided the following locality in the protologue when he described *Photinialongifolia*: “*Loc. nat.* Bengalia orientalis (Griffith, n. 2093.)”. We located two original specimens in K and P, from which the lectotype could be chosen. The information of both duplicates perfectly matched with the locality mentioned by Decaisne. According to Stafleu and Cowan (1976), Decaisne’s specimens are often kept at BR, but many types at P and PC, but also some at G. We, therefore, designate the syntype (P02143220) in P as the lectotype, and the one in K as the isolectotype.

**40. *Mespilusjaponica*** Thunb., Fl. Jap. (Thunberg) 206. 1784. ≡ *Eriobotryajaponica* (Thunb.) Lindl., Trans. Linn. Soc. London 13: 102. 1821. Type: JAPAN. *Thunberg s.n.* (holotype: UPS-THUNB accession no. 11908).

**41. *Symplocosseguinii*** H.Lév., Repert. Spec. Nov. Regni Veg. 10: 431. 1912. ≡ *Eriobotryaseguinii* (H.Lév.) Cardot ex Guillaumin, Bull. Soc. Bot. France 71: 287, in obs. 1924. Type: CHINA. Kouy-Tchéou (Guizhou): Environs de Ou-La-Gay et de Hoang-Ko-Chou, Mars 1899, *J. Séguin & R.P. Bodinier 2617* (**lectotype**, selected by Vidal 1965: 575, first step “type”; **second step, designated here**: E[barcode E00011359]!; isolectotypes: P[barcode P02143232]!, P[barcode P02143233]!). *s. loc.*, 9 April 1898, *J. Séguin & R.P. Bodinier 2262* (syntypes: E[barcode E00011332]!, P[barcode P02143234]!). [image of lectotype available at http://data.rbge.org.uk/herb/E00011359] [note 1]

= *Eriobotryapseudoraphiolepis* Cardot, Notul. Syst. (Paris) 3: 371. 1918. nom. superfl. [note 2]

Note 1: Léveillé (1912) mentioned two gatherings (*J. Séguin 2262, 2617*) in the protologue when describing *Symplocosseguinii*, so they are syntypes. Vidal (1965) selected the gathering (*J. Séguin 2617*) kept at E and P as the lectotype [first-step]. We located three specimens of this gathering, one is in E and the other two in P. According to Stafleu and Cowan (1979), all of the Léveillé’s type specimens have been purchased by E in 1919. We designate the duplicate (E00011359) of *J. Séguin 2617* kept at E as the lectotype [second-step].

Note 2: Cardot (1918) described *Eriobotryapseudoraphiolepis* with the following locality in the protologue: “Kouy-Tcheou: environs de Ou-la-gay et de Hoang-ko-chou [*Séguin et Bodinier*, 1898 et 1899; nos 2262 et 2617]”. It definitely included the type of *Symplocosseguinii* H.Lév. (1912). So *E.pseudoraphiolepis* is a nomenclaturally superfluous name when it was published (Turland et al. 2018: Art. 52.1).
